# The effect of Zexie decoction on vestibular and auditory function in DDAVP-induced endolymphatic hydrops of Guinea pigs

**DOI:** 10.3389/fneur.2025.1430522

**Published:** 2025-02-24

**Authors:** Renlong Ji, Yanchang Xu, Kang Li, Wen Jiang, Yuan Li, Jianwei Zeng, Wei Li, Caiji Wang, Zeqi Zhao, Yalan Li, Naveena Konduru, Wen Liu, Yuehua Qiao, Xuanyi Li

**Affiliations:** ^1^Department of Otorhinolaryngology-Head and Neck Surgery, Affiliated Hospital of Xuzhou Medical University, Xuzhou, China; ^2^Institute of Audiology and Balance Science, Xuzhou Medical University, Xuzhou, China; ^3^Artificial Auditory Laboratory of Jiangsu Province, Xuzhou Medical University, Xuzhou, China; ^4^Department of Radiology, Affiliated Hospital of Xuzhou Medical University, Xuzhou, China; ^5^Department of Otolaryngology Head and Neck Surgery, Gulou Hospital Affiliated to Medical College of Nanjing University, Nanjing, China; ^6^Key Laboratory of New Drug Research and Clinical Pharmacy, Xuzhou Medical University, Xuzhou, China

**Keywords:** Meniere’s disease, endolymphatic hydrops, vestibular evoked myogenic potentials, vestibular function, Ze Xie decoction

## Abstract

**Objective:**

To investigate the effects of Zexie decoction on vestibular and auditory function in guinea pigs with endolymphatic hydrolysis induced by desmopressin. Methods: Sixty guinea pigs were randomly and evenly divided into four groups, each group has 15 guinea pigs: normal control group, DDAVP group, DDAVP modeling combined with Zexie Decoction group, and DDAVP combined with Double Zexie group. At 7 and 14 days, bone-conducted cervical vestibular evoked myogenic potential tests, auditory brainstem responses, and distortion-product otoacoustic emissions were conducted on each group of guinea pigs to evaluate their vestibular and auditory function quantitatively. After functional testing, the outer hair cells were observed by scanning electron microscope. On day 14, one guinea pig was randomly selected from both the normal control group and DDAVP group to verify the successful establishment of the model using gadolinium-enhanced magnetic resonance imaging of the inner ear.

**Results:**

We conducted BC-cVEMP, ABR, and DPOAE tests on guinea pigs, and the results showed that DDAVP did affect vestibular function and hearing in guinea pigs. Analyses were performed from those results that were statistically significant, Zexie Decoction improved DDAVP-induced vestibular dysfunction and hearing loss dose-dependently, though complete reversal was not achieved. About scanning electron microscopy, outer hair cells of the DDAVP group showed significant loss and cilia lodging, however, treatment with Zexie decoction can alleviate the loss of outer hair cells and the lodging of cilia. When the outer hair cells were exposed to DDAVP for a long time, the improvement effect of Zexie decoction was not as obvious as before.

**Conclusion:**

The extent of improvement correlates with the concentration and dosage of Zexie Decoction. Even at double the dosage, Zexie Decoction only partially mitigates the decline in vestibular and auditory function induced by DDAVP, falling short of complete reversal.

## Introduction

Meniere’s disease (MD) is an idiopathic inner ear disorder characterized by recurrent episodes of vertigo, tinnitus, a sensation of pressure or fullness in the ear, and fluctuating sensorineural hearing loss as its primary clinical manifestations (1). The exact mechanism behind the onset of Ménière’s disease remains unclear. Since the discovery of endolymphatic hydrops (EH) in the temporal bone of patients by Hallpike and Cairns in 1938 (2), researchers commonly regard EH as its primary pathological manifestation. Several researchers have identified the saccule as the primary site of noticeable endolymph accumulation, followed by the cochlea, utricle, and semicircular canals, in both animals (3) and humans (4). Presently, the diagnosis of Ménière’s disease heavily relies on clinical presentation, imaging examinations, and auditory tests, which aid in its identification. As for treatment, the primary focus is on managing vertigo attacks, often utilizing systemic diuretics to alleviate the severity of EH (5). Zexie Decoction, a traditional Chinese medicinal formula, traces its roots back to the classical text ‘Jin Kui Yao Lue’, or ‘Synopsis of the Golden Chamber’. This ancient manuscript is revered within the realm of traditional Chinese medicine for its comprehensive exploration of herbal remedies and treatments. The core components of Zexie Decoction are Zexie (Alisma rhizome) and Baizhu (white atractylodes rhizome). Alisma rhizome, derived from the Alisma plant, is celebrated for its potent diuretic qualities. It works by promoting the elimination of excess fluids from the body, thus playing a crucial role in the regulation of water metabolism. White atractylodes rhizome, on the other hand, is sourced from the atractylodes macrocephala plant. It complements alisma rhizome by reinforcing the body’s ability to manage fluid balance, further enhancing the formula’s effectiveness in treating conditions related to fluid imbalance.

The synergy between these ingredients endows Zexie Decoction with remarkable diuretic and edema-reducing capabilities. It’s precisely this combination that allows the decoction to efficiently remove unnecessary body fluids, making it a staple in treatments aimed at reducing swelling and alleviating the discomfort associated with excess water retention. In the broader context of traditional Chinese medicine theory, there’s a well-acknowledged link between the health of the kidneys and the ears. The kidneys are believed to play a pivotal role in water metabolism and, by extension, in maintaining the equilibrium of bodily fluids. This balance is crucial for ear health, as imbalances can lead to symptoms such as dizziness and vertigo. Zexie Decoction, with its potent diuretic effect, is therefore commonly employed in clinical settings to treat these symptoms. Its application is rooted in the belief that by regulating kidney function and, consequently, the body’s fluid balance, it can effectively mitigate the issues leading to dizziness and vertigo, showcasing its significant therapeutic potential in this domain (6, 7).

While some English literature reports on the effects of Zexie Decoction on various body organs (8, 9), specify chinese literature documents its efficacy in alleviating vertigo attacks among Meniere’s disease patients. However, there remains a lack of extensive research elucidating the mechanisms by which Zexie Decoction operates within the inner ear to manage vertigo attacks. Presently, there’s a scarcity of corresponding fundamental experiments to objectively evaluate whether Zexie Decoction effectively ameliorates the decline in vestibular and auditory functions associated with EH. In this experiment, we utilized a desmopressin (DDAVP)-induced guinea pig model of EH as the primary research subject. The successful modeling of EH was verified using inner ear-enhanced Gd MRI. With Zexie Decoction as the stimulating factor, the evaluation of Zexie Decoction’s impact on the vestibular and auditory functions of guinea pigs with EH was conducted utilizing bone-conducted vestibular evoked myogenic potentials (BC-VEMP), auditory brainstem evoked potentials (ABR), and distortion-product otoacoustic emissions (DPOAE) as detection methods, then, the outer hair cells were observed by scanning electron microscope.

## Materials and methods

### Experimental animals

Sixty albino red-eyed guinea pigs, weighing between 250 to 350 grams, were chosen for the study. All animals demonstrated sensitive ear reflexes, intact eardrums, and exhibited no signs of middle ear effusion upon otoscopic examination. Furthermore, the recorded ABR thresholds were within the range of 35 dB SPL.

### Main reagents and instruments

Acetic acid desmopressin injection. (deamino-Cys1,D-Arg8-Vasopressin, DDAVP, Aladdin Corporation, Batch Number: D123296), Gadopentetate dimeglumine injection, Ear endoscope, auditory brainstem response tester and otoacoustic emissions. (Neuro-Audio, Neurosoft Corporation), Broadband Acoustic Impedance Tester(Mimosa, Danish International Auditory/Interacoustics company), Electrode needle (diameter 0.25 mm, length 25 mm), 1 mL syringe, 3 T MRI scanner (Verio, Siemens Medical, Erlangen, Germany), and 32-channel phased-array coil.

### Zexie decoction

The rhizoma alismatis (origin: Meishan, Sichuan; batch number: A220219), Atractylodes macrocephala (origin: Huaibei, Anhui; batch number: A220410), the implementation standards are: “Chinese Pharmacopoeia” 2020 edition of the first part. The production method of Zexie Decoction in the book “Jin Kui Yao Lue” is as follows: 5 taels of rhizoma alismatis, 2 taels of Atractylodes macrocephala, the ratio of the two is 5:2. According to the conversion rate of 1 tael in ancient times to 10 g today, adults should take a total of 70 g of the two medicinal materials daily. According to the conversion ratio of gastric gavage between humans and guinea pigs, guinea pigs should be given a daily gastric gavage of 0.7 g/100 g. According to the proportions in the original formula, two herbal medicines are mixed and soaked in water for 1 h, boiled and cooked for 30 min, and the herbal liquid is filtered. The herbal residue is boiled again with water for 30 min, and the herbal liquid is filtered again. The two herbal liquids are mixed and concentrated into a solution containing 0.7 g/mL of crude herbs.

To study the effect of high-concentration Zexie Decoction on vestibular function, a double-concentration method was used to prepare a solution of double-concentration Zexie Decoction with a concentration of 1.4 g/mL.

## Experimental method

Sixty guinea pigs were randomly and evenly divided into four groups, with 15 animals in each group: normal control group, DDAVP group, DDAVP modeling combined with Zexie Decoction group (Zexie group), and DDAVP modeling combined with Double Zexie group (Double Zexie group).

The normal control group was established by administering sterile saline via intraperitoneal injection, while the other three groups received intraperitoneal injections of DDAVP at a dosage of 10 μg/mL/kg daily for 14 consecutive days (6). The normal control group and the DDAVP group did not received gavage.

The Zexie group and the Double Zexie group: Based on intraperitoneal injection of desmopressin (10 μg/mL/kg), the guinea pigs were fed with Zexie Decoction (0.7 g/mL) and double dose of Zexie Decoction (1.4 g/mL) for 14 consecutive days.

### ABR test

**Figure 1:**
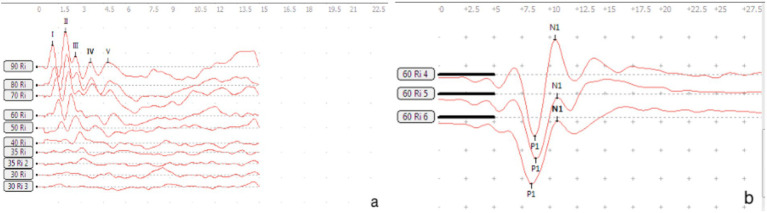
Waveforms of BC-VEMP and ABR detection in guinea pigs.

Testing was performed at two separate time points, 7 and 14 days, guinea pigs received intraperitoneal injections of pentobarbital sodium (30 mg/kg) and were positioned prone in a soundproof room. Silver needle electrodes were placed in the calvarium and the ipsilateral retroarticular region. A rubber sound tube was inserted into the external auditory canal for stimulation. Analysis and recording were performed using the Neuro-Audio otoacoustic emission and evoked potential amplifier by Russia Reso Company. Short sound stimulation of 0.1 ms within the frequency range of 100 Hz-2000 Hz was applied, repeating 21 times per millisecond with an average of 300 scans. ABR threshold determination began at 90 dB SPL, decreasing by 10 dB until the waveform was no longer detectable. VEMP assessments were conducted under anesthesia following ABR tests (refer to Figure 1).

### BC-cVEMP test

**Figure 2:**
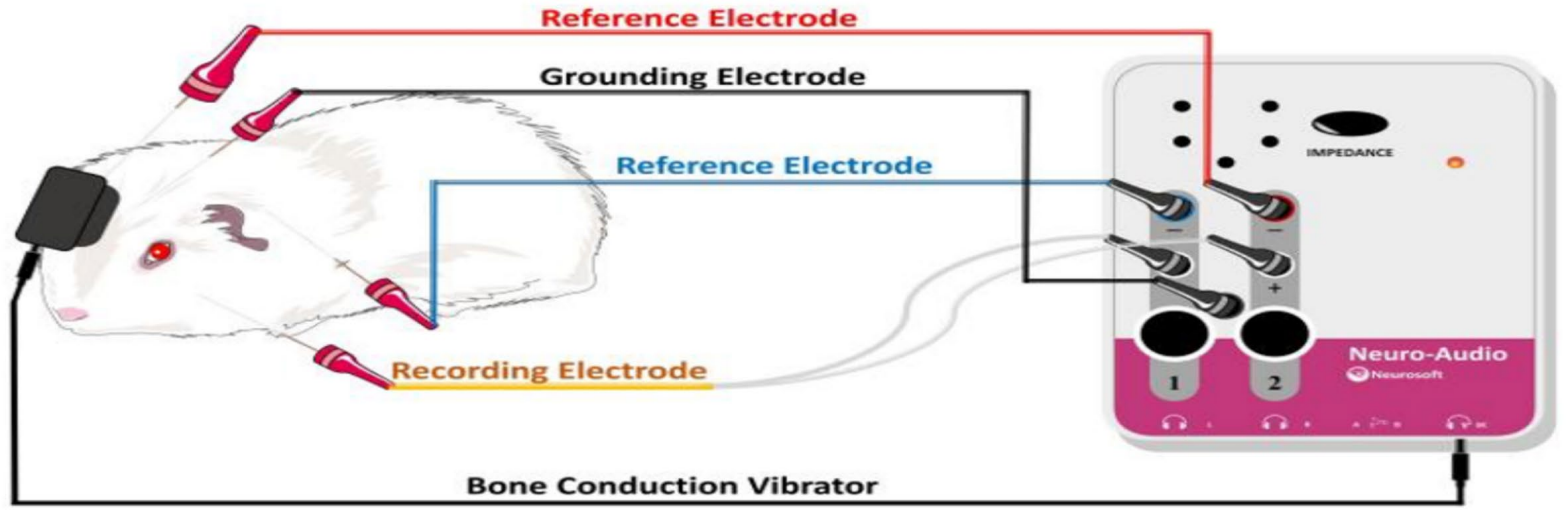
Schematic representation of BC-VEMP detection in guinea pigs.

Testing was performed at two separate time points 7 and 14 days, the BC-cVEMP experimental method used in this study was based on the method established by Curthoys et al. (10, 11) and Yang et al. (12). Each guinea pig underwent anesthesia via intraperitoneal injection of 3% pentobarbital sodium (30 mg/kg) prior to VEMP measurements. The assessments were conducted in a soundproof shielded room, employing silver electrode needles (diameter 0.25 mm, length 25 mm). During cVEMP examination, the recording electrode was precisely positioned on the sternocleidomastoid muscle of the guinea pig. The ground electrode was placed at the midline between the ears on the skull, while the reference electrode was positioned behind the ear, ensuring a depth of 5–10 mm and interelectrode resistance of less than 5kΩ. For optimal muscle tension, the guinea pig’s head was turned to the opposite side after stimulation, engaging the ipsilateral cervical flexor muscles. The Russian Reso otoacoustic emission and evoked potential amplifier (Neuro-Audio type) were utilized for analysis and recording. Stimulation involved 500 Hz tone burst stimulation, delivering sound and recording on the same side. Parameters included a scanning time of 50 ms, a bandpass filter range of 1-500 Hz, 49-fold superposition, a stimulus intensity of 95 dB SPL, and a stimulation frequency of 3 times/s. Each intensity was tested 2–3 times for accuracy. The first negative wave in the waveform is P1, and the first positive wave is N1 (13). Latency and amplitude were meticulously recorded, with the average value considered as the result. All experiments were conducted in a sound-insulated environment (Figures 1, 2).

### DPOAE test

Testing was performed at two separate time points, 7 and 14 days, guinea pigs were anesthetized with intraperitoneal injection of pentobarbital sodium (30 mg/kg). The test was performed in a soundproof shielding room with a rubber sound tube placed in the external auditory canal. The test frequencies were 750, 1,000, 2000, 4,000, 6,000, 8000HZ, and the elicited criteria were SNR ≥ 6 dB and DP amplitude ≥-10 dB SPL.

### Gadolinium contrast-enhanced magnetic resonance imaging of the inner ear

At 14 days, one guinea pig was randomly selected from the normal control group and the DDAVP group, after anesthetizing guinea pigs with intraperitoneal pentobarbital sodium injection (30 mg/kg), MRI scanning was conducted using a 3 T Verio magnetic resonance device by Siemens Healthcare, Erlangen, Germany. A 32-channel phased-array head coil was used 24 h post bilateral tympanic injection of Gd contrast agent (Gadopentetate dimeglumine injection). MRI images were obtained using T2 space, 3D Real-IR, and 3D-FLAIR sequences. For the 3D Real-IR sequence: voxel size was 0.4 × 0.4 × 0.8 mm, scan time 14 min, TR 9000 ms, TE 181 ms, TI 1730 ms, slice thickness 0.80 mm, FOV 160 × 160 mm, and matrix size 3,300 × 918. Regarding the 3D-FLAIR sequence: voxel size 0.7 × 0.7 × 0.6 mm, scan time 6 min, TR 6000 ms, TE 387 ms, TI 2100 ms, slice thickness 0.60 mm, echo train length 173, FOV 220 × 220 mm, and matrix size 1701 × 810.

### Scanning electron microscopy

At 7 and 14 consecutive days, four guinea pigs were randomly selected from each of the four groups, guinea pigs were anesthetized with 3% pentobarbital, decapitated, and their cochleae rapidly excised and immersed in 2.5% glutaraldehyde at 4°C. For basement membrane dissection, under a dissecting microscope, cochlear structures were manipulated to allow perfusion of fixative, flush perilymph, and enable removal of the basilar and vestibular membranes. The isolated basilar membranes were then fixed in 2.5% glutaraldehyde. The samples underwent a cleaning step with saline, followed by pre-fixation in 2–3% glutaraldehyde (pH 6.8–7.2) at 4°C for over two hours. This was followed by three buffer rinses and post-fixation in 1% osmium tetroxide at 4°C for 1–2 h. Further rinsing involved three cycles with pure water, gradient dehydration using increasing concentrations of ethanol or acetone (50 to 95%), and final treatment with absolute ethanol. Samples were then critical point dried, adhered to a conductive double-sided tape on the sample table with the observation surface upwards, surface-treated by ion sputtering, and finally examined under a scanning electron microscope.

### Statistical methods

**Figure 3:**
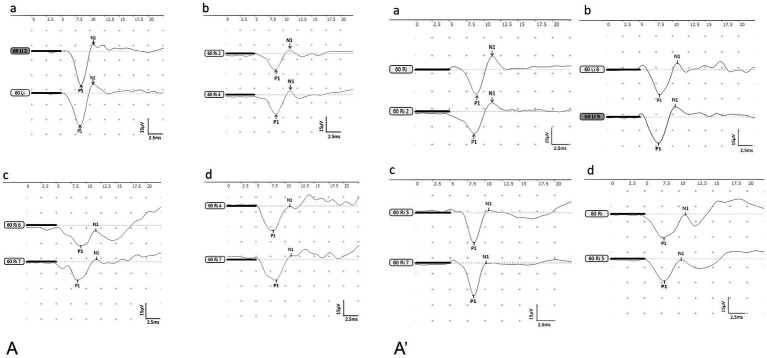
The waveforms of BC-cVEMP **(A)**: the control group, **(B)**: the DDAVP group, **(C)**: the Zexie group, **(D)**: the Double Zexie group. **(A)**: the waveforms of the four groups at 7 days. **(A’)**: the waveforms of the four groups at 14 days.

SPSS 26.0 statistical software was used to process and analyze the data. All data were expressed as the mean ± standard deviation, the significance level was set at *α* = 0.05, and statistical significance was defined as *p* < 0.05. One-way analysis of variance was used to analyze the data conforming to normal distribution and homogeneity of variance, and the LSD-t test was used for multiple comparisons. The Kruskal-Wallis test was used to compare the data between the two groups if the distribution did not conform to the normal distribution (Figure 3).

## Results

### BC-cVEMP results

DDAVP can cause vestibular dysfunction in guinea pigs, and Zexie decoction has an ameliorating effect on this dysfunction.

**Figure 4:**
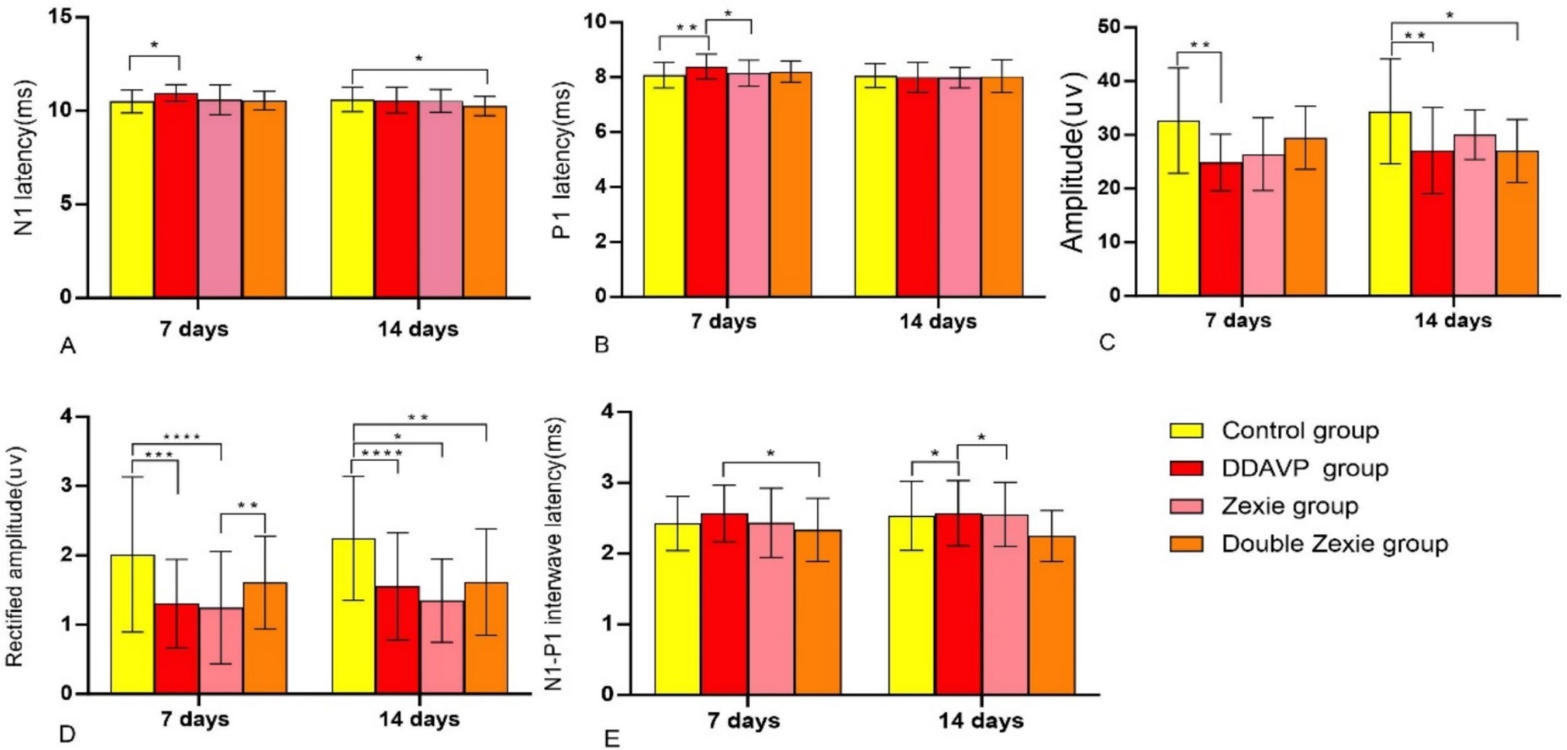
Test result of BC-cVEMP **(A)** After 7 days, the latency of N1 in the DDAVP group was significantly higher than that in the normal control group; after 14 days, the latency of N1 in the normal control group was slightly higher than that in the Double Zexie group (*p* < 0.05). **(B)** At 7 days, the latency of P1 in the DDAVP group was slightly higher than that in the Zexie group, and the latency of P1 in the DDAVP group was significantly higher than that in the normal control group (*p* < 0.05). **(C)** At 7 days, the amplitude in the normal control group was higher than that in the DDAVP group; at 14 days, the amplitude in the normal control group was higher than that in the DDAVP group, and similarly, the amplitude in the normal control group was higher than that in the Double Zexie group (*p* < 0.05). **(D)** At 7 days, the corrected amplitude in the normal control group was higher than that in the DDAVP group; at 14 days, the corrected amplitude in the normal control group was higher than those in the DDAVP group, Zexie group, and Double Zexie group (*p* < 0.05). **(E)** At 7 days, the interval of N1-P1 wave in the Double Zexie group was lower than that in the DDAVP group; at 14 days, compared to the DDAVP group, the interval of N1-P1 in the normal control group showed a slight decrease, while the interval of N1-P1 in the DDAVP group exhibited a minor increase compared to the Zexie group (*p* < 0.05). *means *p* < 0.05, **means *p* < 0.01, ***means *p* < 0.001, and ****means *p* < 0.0001.

After the establishment of the model for 7 consecutive days, statistically significant differences (*p* < 0.05) were observed:

Both N1 and P1 latencies in the DDAVP group were notably higher compared to the normal control group (Figure 4A).
The P1 latency in the DDAVP group exhibited a slight increase when compared to the Zexie group (Figure 4B).
(3) The amplitude and corrected amplitude in the DDAVP group were significantly lower than those in the normal control group (Figures 4C,D).
(4) The N1-P1 wave interval in the Double Zexie group was lower than that in the DDAVP group (Figure 4E).

No statistically significant differences were found in the latency, amplitude, and corrected amplitude of N1 between the DDAVP group and the Zexie group, or between the DDAVP group and the Double Zexie group. Similarly, no significant differences were observed in terms of P1 latency between the normal control group and the DDAVP group, or between the DDAVP group and the Double Zexie group. Additionally, regarding the N1-P1 wave interval, no significant differences were detected between the normal control group and the DDAVP group, or between the DDAVP group and the Zexie group.

When the model was established for 14 consecutive days, the data with statistical differences (*p* < 0.05) were as follows:

1. The N1 latency of the normal control group was marginally higher than that of the Double Zexie group (Figure 4A).
2. The amplitudes in the normal control group were greater than those in the DDAVP group (Figure 4C). Similarly, the normal control group exhibited higher amplitudes compared to the Double Zexie group (Figure 4C).
3. Corrected amplitude values in the normal control group were notably higher than those in the DDAVP group, Zexie group, and Double Zexie group (Figure 4D).
4. The N1-P1 interval of the normal control group displayed a slight decrease compared to that of the DDAVP group, while the N1-P1 interval of the DDAVP group showed a minor increase compared to the Zexie group (Figure 4E). Other parameters did not exhibit statistically significant differences.

**Figure 5:**
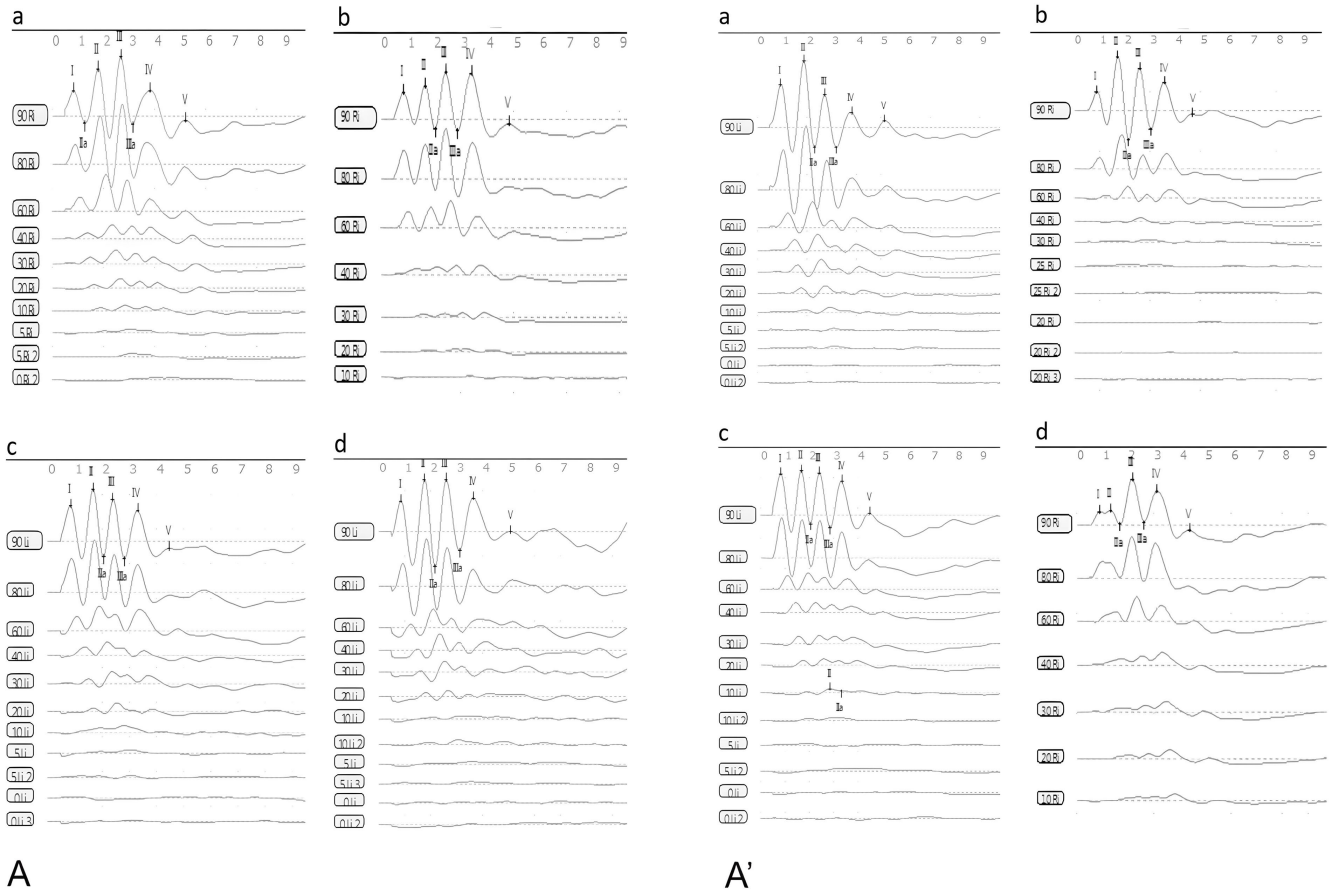
The waveforms of ABR **(A)**: the control group, **(B)**: the DDAVP group, **(C)**: the Zexie group, **(D)**: the Double Zexie group **(A)**: the waveforms of the four groups at 7 days. **(A’)**: the waveforms of the four groups at 14 days.

The aforementioned findings indicate that DDAVP induces a reduction in vestibular function among guinea pigs with EH. Treatment with Zexie decoction shows a subsequent improvement in vestibular function. The double concentration of Zexie decoction demonstrates a more pronounced effect in ameliorating this condition; however, it does not entirely mitigate the impact of DDAVP. Moreover, with prolonged application of DDAVP, the therapeutic efficacy of Zexie decoction appears to diminish accordingly (Figure 5).

### ABR results

DDAVP can cause hearing loss in guinea pigs, and Zexie decoction can improve this hearing loss caused by DDAVP, and it is related to the dosage and application time of Zexie decoction.

**Figure 6:**
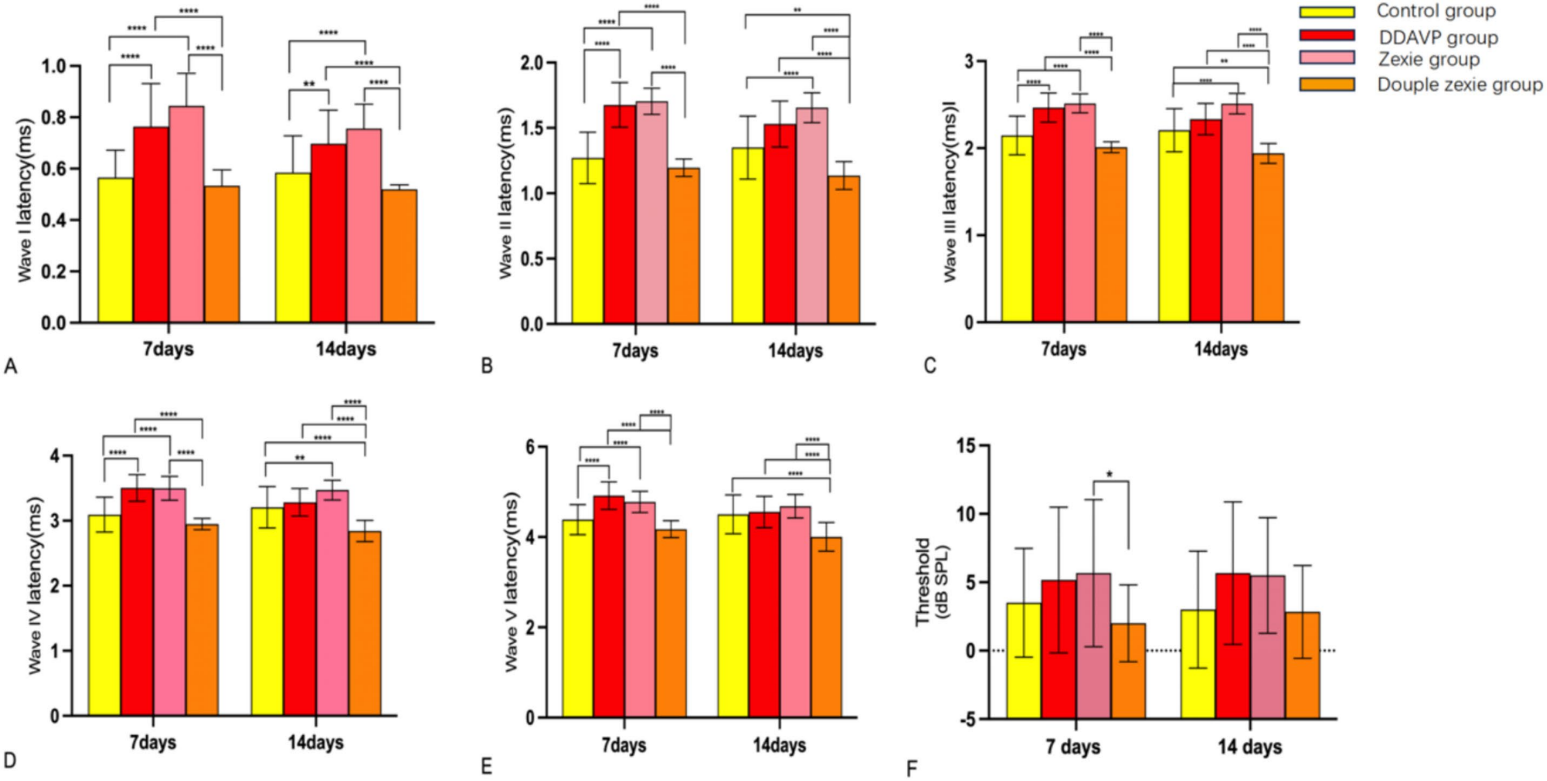
Test result of ABR **(A–E)**: at 7 days, the latency of waves I, II, III, IV, and V in the normal control group was lower than that in the DDAVP and Zexie groups. Additionally, the latency of waves I, II, III, IV, and V in the Double Zexie group was lower than that in the Zexie and DDAVP groups (*p* < 0.05). At 14 days, **(A,B)**: the latency of waves I, II, III, and IV in the normal control group was lower than that in the Zexie group (*p* < 0.05). Additionally, the latency of waves I and II in the normal control group was lower than that in the DDAVP group (*p* < 0.05). **(A–E)**: the latency of waves I, II, III, IV, and V in the Double Zexie group was lower than that in the Zexie and DDAVP groups (*p* < 0.05). The latency of waves III, IV, and V in the normal control group was lower than that in the Double Zexie group (*p* < 0.05). *means *p* < 0.05, **means *p* < 0.01, ***means *p* < 0.001, and ****means *p* < 0.0001.

The statistically significant differences observed after establishing the model for 7 consecutive days were as follows: the latency of waves I, II, III, IV, and V in the normal control group was lower than that in the DDAVP and Zexie groups (Figures 6A–E). Furthermore, the latency of waves I, II, III, IV, and V in the Double Zexie group was lower than that in the Zexie group and the DDAVP group (Figures 6A–E). Additionally, the threshold of the Zexie group was higher than that of the Double Zexie group (Figure 6F). Other parameters did not yield statistically significant differences.

When modeling for 14 consecutive days, statistically significant differences were observed: the latency of waves I, II, III, and IV in the normal control group was lower than that in the Zexie group (Figures 6A–D). Additionally, the latency of waves I and II in the normal control group was lower than that in the DDAVP group (Figures 6A,B). Moreover, the latency of waves I, II, III, IV, and V in the Double Zexie group was lower than that in the Zexie group and the DDAVP group (Figures 6A–E). Notably, the latency of waves III, IV, and V in the normal control group was lower than that in the Double Zexie group (Figures 6C–E). Other parameters did not exhibit statistically significant differences.

### DPOAE results

Statistically significant differences were noted in amplitude, noise, signal-to-noise ratio (SNR), and phase after continuous modeling for both 7 and 14 days at a specific frequency in the DPOAE measurement.

#### Comparison of amplitude changes

**Figure 7:**
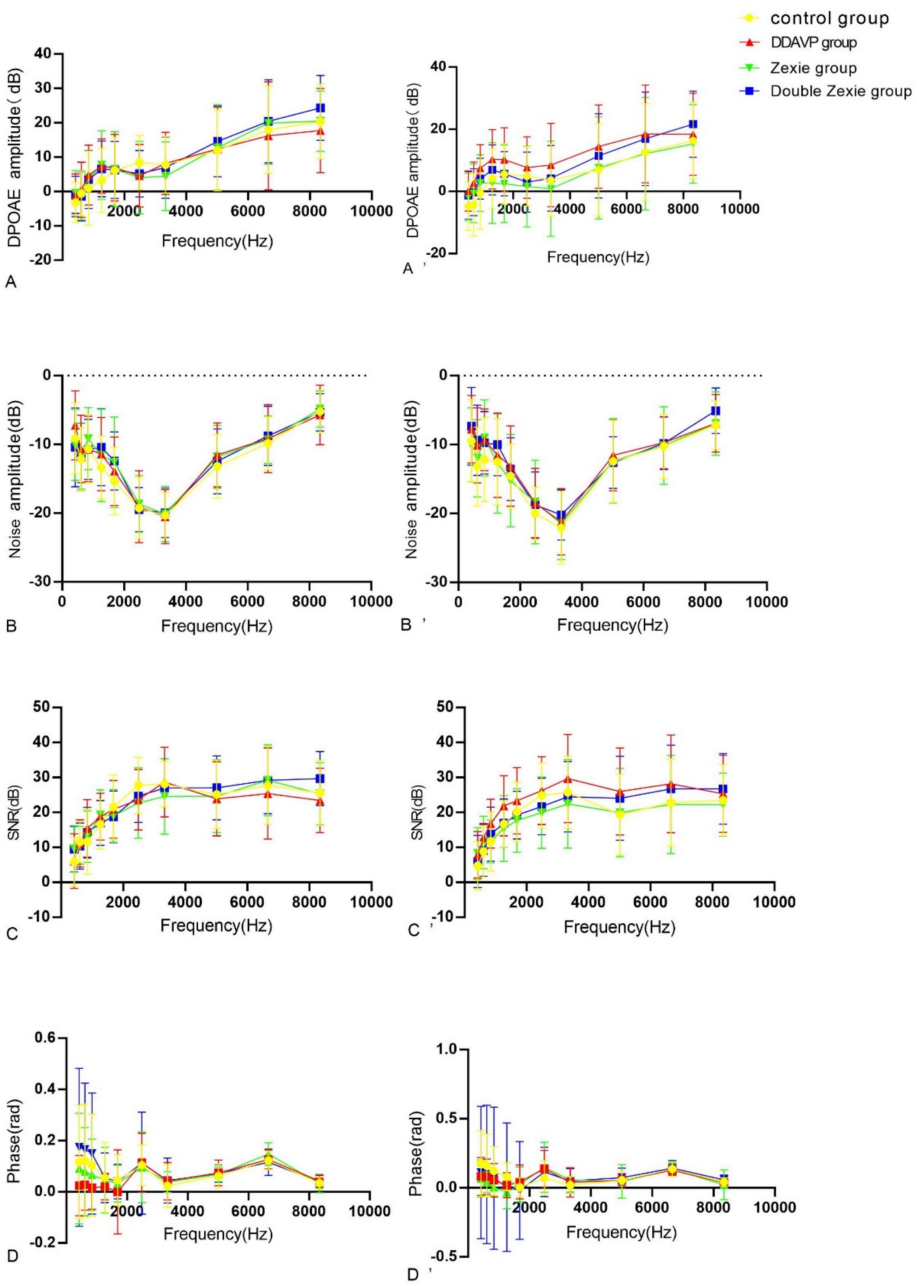
Test result of DPOAE **(A–D)** are the amplitude, noise, signal-to-noise ratio (SNR) and phase of DPAOE for 7 consecutive days. **(A’–D’)** represents the amplitude, noise, signal-to-noise ratio (SNR) and phase of DPAOE for 14 consecutive days. **(A’)** At 14 days, at 422 Hz and 609 Hz, the amplitude in the normal control group was higher than that in the DDAVP group (*p* < 0.05). Conversely, at 844 Hz and 1,688 Hz, the amplitude in the normal control group was lower than that in the DDAVP group (*p* < 0.05). Notably, at 1688 Hz, the amplitude of the DDAVP group was higher than that of the Zexie and Double Zexie groups (*p* < 0.05). **(B)** At 7 days, the noise level of the Double Zexie group at 422 Hz was higher than that of the Zexie group (*p* < 0.05). **(B’)** After 14 days of continuous modeling, at 844 Hz, the noise level of the normal control group was significantly lower than that of the DDAVP and Zexie groups (*p* < 0.05) **(C)** After 7 days of continuous modeling, at 8344 Hz, the signal-to-noise ratio of the DDAVP group was lower than that of the Double Zexie group (*p* < 0.05). **(C’)** After 14 days of continuous modeling, at 1266 Hz, 1,688 Hz, and 2,484 Hz, the DDAVP group showed a higher signal-to-noise ratio compared to the Zexie group (*p* < 0.05). At 2484 Hz, the signal-to-noise ratio of the normal control group was significantly higher than that of the Zexie group (*p* < 0.05). **(D)** After 7 days of continuous modeling, at 609 Hz, the phase of the Zexie group was lower than that of the Double Zexie group (*p* < 0.05); at 6656 Hz, the phase of the Zexie group was higher than that of the Double Zexie group (*p* < 0.05). **(D’)** After 14 days of continuous modeling, at 609 Hz, 844 Hz, and 1,266 Hz, the phase of the normal control group was higher than that of the Zexie group (*p* < 0.05); at 1266 Hz, the phase of the normal control group was higher than that of the DDAVP group, while the phase of the Double Zexie group was higher than that of the Zexie group (*p* < 0.05).

After 7 days of modeling, no significant differences were observed across all frequencies. However, after 14 days of continuous modeling, statistically significant differences emerged. Specifically, at 422 Hz and 609 Hz, the normal control group exhibited higher amplitude compared to the DDAVP group (Figure 7A’). Conversely, at 844 Hz and 1,688 Hz, the normal control group showed lower amplitude than the DDAVP group (Figure 7A’). Notably, at 1688 Hz, the DDAVP group displayed higher amplitude compared to both the Zexie group and the Double Zexie group (Figure 7A’). All other frequencies did not exhibit statistically significant differences.

#### Comparison of DPOAE noise changes

After 7 days of continuous modeling, a statistically significant difference was observed in the noise level of the DDAVP group at 422 Hz, which was higher compared to both the Double Zexie group and the Zexie group (Figure 7B). However, no other frequencies exhibited statistically significant differences. Following 14 days of continuous modeling, at 844 Hz, the noise level of the normal control group was significantly lower than that of both the DDAVP group and the Zexie group (Figure 7B’). All other frequencies did not show statistically significant differences.

#### Comparison of DPOAE signal to noise ratio SNR changes

After 7 days, a statistically significant difference was observed at 8344 Hz, where the DDAVP group exhibited a lower signal-to-noise ratio compared to the Double Zexie group (Figure 7C). After 14 days, significant differences were found at 1266 Hz, 1,688 Hz, and 2,484 Hz, with the DDAVP group showing a higher signal-to-noise ratio compared to the Zexie group (Figure 7C’). At 2484 Hz, the signal-to-noise ratio of the normal control group was significantly higher than that of the Zexie group (Figure 7C’). All other frequencies did not show statistically significant differences.

#### Comparison of DPOAE phase changes

After 7 days, statistically significant differences were observed: at 609 Hz, the phase of the Zexie group was lower than that of the Double Zexie group (Figure 7D); at 6656 Hz, the phase of the Zexie group was higher than that of the Double Zexie group (Figure 7D), while the amplitude, noise, signal-to-noise ratio, and phases at other frequencies were not statistically significant. After 14 days, significant differences were found: at 609 Hz, 844 Hz, and 1,266 Hz, the phase of the normal control group was higher than that of the Zexie group (Figure 7D’); at 1266 Hz, the phase of the normal control group was higher than that of the DDAVP group, while the phase of the Double Zexie group was higher than that of the Zexie group (Figure 7D’), and the amplitude, noise, signal-to-noise ratio, and phases at other frequencies were not statistically significant.

### Contrast-enhanced Gd MRI of the inner ear

DDAVP can cause endolymphatic hydrops in the guinea pig cochlea.

24 h after local administration of gadopentetate dimeglumine, the contrast agent enters the perilymph through the round window membrane but does not penetrate the endolymph. This results in the perilymph appearing as high signal on MRI-real IR sequences due to the presence of the contrast agent, while the endolymph, lacking the agent, shows as low signal. Since the inner ear is encased in the bony labyrinth, a closed space, the presence of endolymphatic hydrops (EH) can be determined by observing the signal ratio between the perilymph and endolymph.

**Figure 8:**
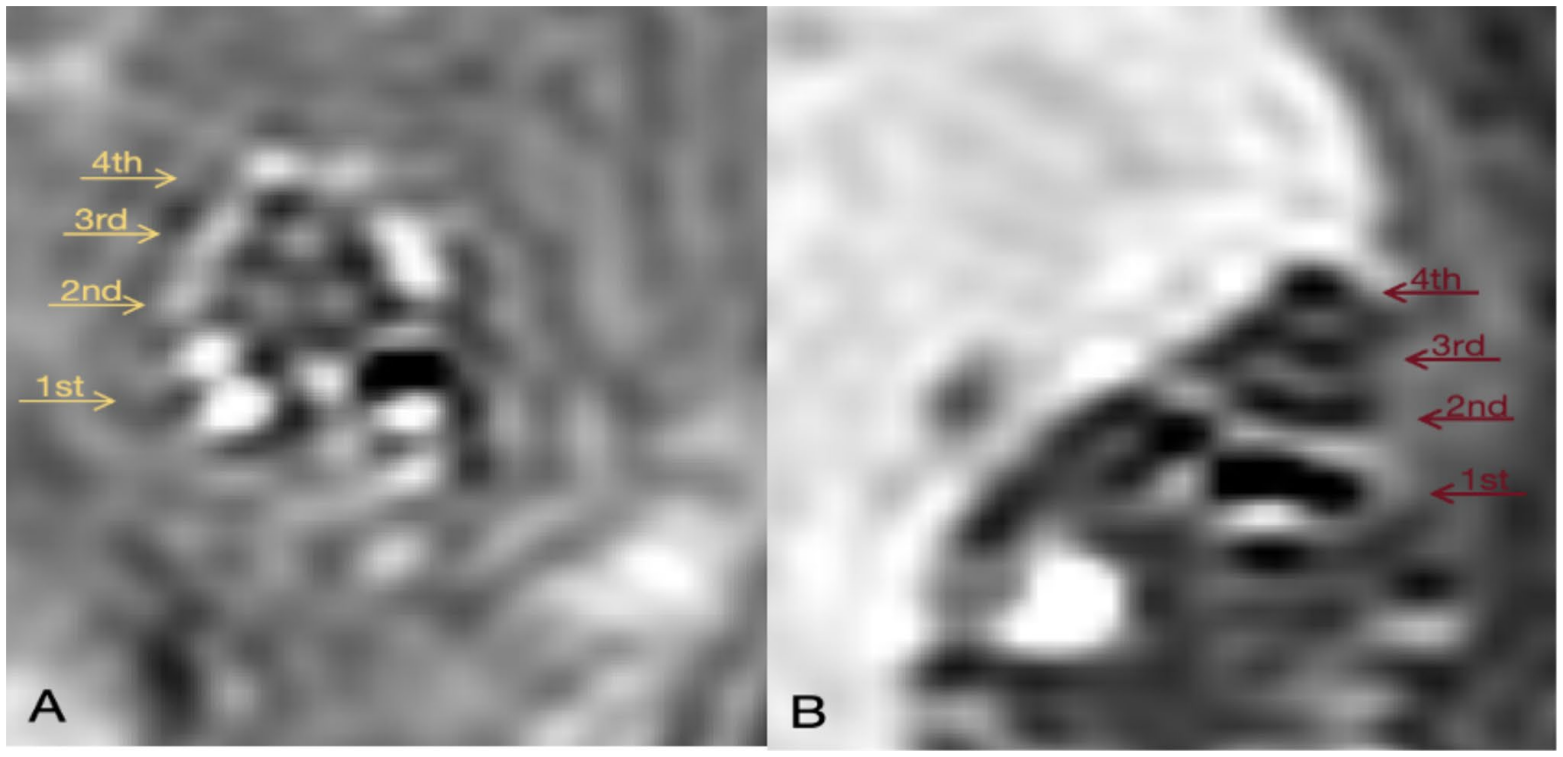
GD-enhanced MR imaging of the inner ear in Guinea Pigs from the Control and DDAVP Groups. **(A)** 3D Real-IR imaging illustrating the cochlea in the normal control group. This panel depicts a healthy cochlea with distinguishable signals in the perilymphatic and endolymphatic spaces, exhibiting a normal proportion between them. **(B)** 3D Real-IR imaging displaying the cochlea in the DDAVP group. In this panel, a significant enlargement of the endolymphatic space is evident, accompanied by a relative reduction in the perilymph space, indicative of the presence of EH. 1st represents the basal turn; 2nd indicates the second turn; 3rd indicates the third turn; 4th is the apical return.

On the 14th day following DDAVP administration, a pre-injection of Gadopentetate dimeglumine was administered into the tympanic cavity 24 h prior to performing GD-enhanced MRI scans of the guinea pigs’ inner ears. The MRI imaging revealed evident EH in the inner ears of guinea pigs belonging to the DDAVP group (Figure 8B), confirming the successful replication of the model.

### Scanning electron microscopy

Zexie decoction can improve the loss of outer hair cells and cilia lodging to some extent, but it cannot be completely reversed.

**Figure 9:**
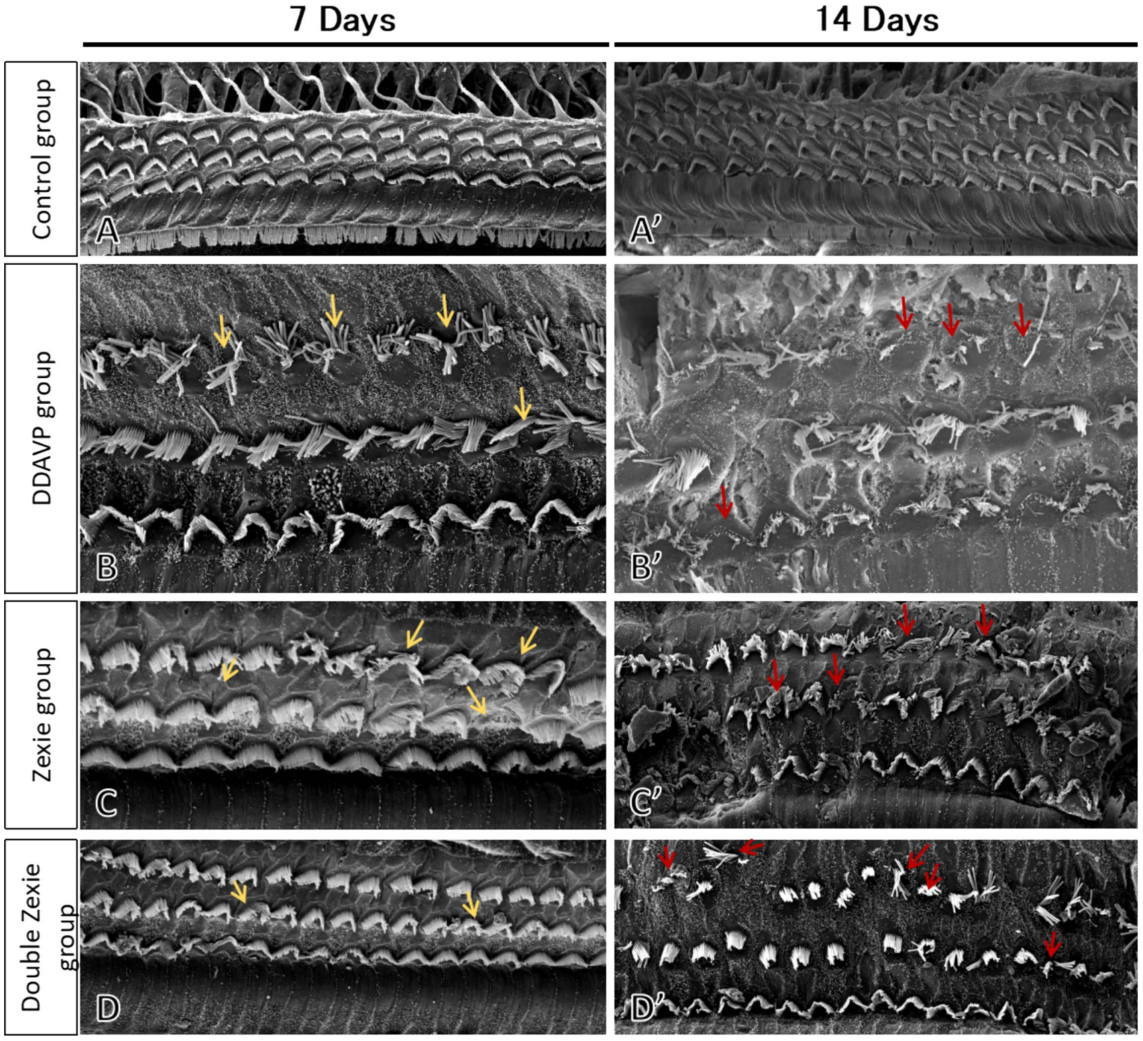
Scanning electron microscopy of the guinea pig cochlea SEM image of the blank control group at 7 days **(A)** and 14 days **(A’)** showed that the outer hair cells were intact and the cilia were neat without loss or collapse. **(B)** At 7 days, the DDAVP group showed obvious collapse and loss of the cilia in the outer hair cells. At 14 days, the DDAVP group exhibited severe degeneration of outer hair cells and significant displacement and loss of cilia **(B’)**. **(C)** The Zexie decoction group at 7 days exhibited displacement and loss of cilia in outer hair cells, showing improvement compared to the DDAVP group **(C)**; at 14 days, the group demonstrated significant displacement and loss of cilia, along with loss of outer hair cells, yet still marked an improvement over the DDAVP group **(C’)**. **(D)** At 7 days post-modeling, the majority of outer hair cells in the double-dose Zexie decoction group appeared normal, albeit with sporadic ciliary dislocation and loss observed **(D)**. By day 14, this group exhibited notable outer hair cell attrition, with apparent ciliary dislocation and loss in the residual cells **(D’)**.

Electron microscopy results revealed that the cilia of outer hair cells in the blank control group were completely intact, without any missing or collapsed cilia (Figures 9A,A’). After 7 days, in the DDAVP group, there was a noticeable collapse and loss of cilia in outer hair cells (Figure 9B), whereas in the Zexie decoction group, these conditions were slightly improved compared to the DDAVP group (Figure 9C). In the double-dose Zexie decoction group, the cilia of most outer hair cells appeared normal, but there were still instances of cilia collapse and loss observed (Figure 9D). After continuous modeling for 14 days, the outer hair cells in the DDAVP group exhibited severe collapse and loss of cilia (Figure 9B’). While the Zexie decoction group also showed significant cilia collapse and loss, the extent was markedly less severe than in the DDAVP group (Figure 9C’). Despite using a double dose of Zexie decoction, cilia loss was still evident, with residual hair cells also displaying missing and collapsed cilia (Figure 9D’).

## Discussion

While some studies have indicated that the degree of EH remains unchanged even after effective vertigo control in Meniere’s patients (14, 15), the understanding of EH and its formation mechanism remains a central focus in current research, considering its role as the characteristic pathological manifestation of Meniere’s disease. To manage severe Meniere’s disease, treatments like intratympanic injections of glucocorticoids or gentamicin, and semicircular canal packing are effective for controlling vertigo attacks. However, most patients experience mild-to-moderate symptoms. Thus, developing effective drug treatments for these patients to reduce vertigo attacks is crucial. Additionally, Meniere’s disease patients often face ongoing reduced vestibular function and hearing loss, even when vertigo attacks subside. While hearing aids can help address hearing loss when needed, options are limited. Vestibular rehabilitation offers a solution for diminished vestibular function after remission, though it presents its challenges. Despite these difficulties, seeking effective drug treatments for these issues remains important.

This study explores the efficacy of Zexie decoction, a herbal remedy derived from ‘Jin Kui Yao Lue’ (Synopsis of the Golden Chamber). Comprising Rhizoma alismatis and Atractylodes macrocephala, this traditional Chinese medicine (TCM) formulation is believed to exhibit diuretic properties, aiding in eliminating excess body fluids and regulating water metabolism. In traditional Chinese medicine clinical practice, Zexie decoction’s diuretic effects are commonly applied to address conditions associated with edema. Moreover, traditional Chinese medicine theory underscores the relationship between kidney function and the ears. This theory, known as ‘kidney opening the ear,’ posits an interconnection between kidney metabolism and ear health. Consequently, Zexie decoction is frequently utilized in clinical treatments for vertigo due to its significant impact. While existing Chinese literature highlights the positive impact of Zexie decoction in treating Meniere’s disease, there remains a dearth of fundamental experimental evidence to confirm its efficacy in improving vestibular and cochlear function.

Currently, there are various methods to induce guinea pig models of endolymphatic hydrops, such as endolymphatic sac obstruction surgery, antidiuretic hormones, and lipopolysaccharide (LPS). Surgical models, although capable of inducing EH by obstructing the endolymphatic sac, are being gradually replaced by medication-induced models due to the significant surgical trauma involved and the discrepancy between their mechanisms and the etiologies observed in clinical patients with endolymphatic hydrops. LPS is another well-established method for inducing severe endolymphatic hydrops while also accounting for immune and inflammatory responses. However, as LPS is an endotoxin derived from the metabolic byproducts of Gram-negative bacteria, and Ménière’s disease patients typically lack a history of suppurative otitis media, the mechanism of LPS-induced membranous labyrinth hydrops significantly differs from the pathogenesis of Ménière’s disease in clinical settings (3, 16). Among medication-induced methods, DDAVP, an antidiuretic hormone, is the most commonly used approach. Based on our team’s previous research (6, 7), we also utilized the DDAVP-induced model of membranous labyrinth hydrops to evaluate the therapeutic effects of Zexie Decoction.

We utilized BC-cVEMP tests, ABR, and DPOAE as means of quantitatively evaluating the vestibular and auditory functions across different groups. Additionally, to explore the relationship between dosage and efficacy, we included a group treated with double the dose of Zexie decoction.

VEMP are responses generated by stimulating the vestibular otolithic organ with robust sounds, eliciting reflexive muscle contractions through a specific pathway. This response is a manifestation of the vestibular reflex, following a pathway from the saccule to the inferior vestibular nerve, vestibular nucleus, lateral vestibular nucleus, lateral vestibulospinal tract, and ultimately activating the sternocleidomastoid muscle. Electrodes placed on the skin surface record this electrical response, which can be categorized into air-conducted acoustic stimulation-induced VEMP or those evoked by bone-conducted vibration stimulation. The latter is thought to activate specific afferent neurons through sound or vibration, originating from specialized type I receptors in distinct regions of the otolithic membrane. These bone-conducted receptors transmit signals from the cystic macula to the ipsilateral sternocleidomastoid muscle, leading to rapid suppression of myogenic potential in these muscles with a short latency (17). The purpose of this study was to observe the effect of Zexie decoction on vestibular function in guinea pigs with endolymphatic hydrolysis by BC-cVEMP. BC-cVEMP, utilized here, involve inducing linear acceleration of the mastoid process through compression and shear wave transmission via bone-conducted vibration stimulation applied directly to the skull using bone-conduction headphones. This method circumvents the outer and middle ear, directly impacting the vestibular organ within the inner ear, selectively activating type I vestibular hair cells and irregular otolith afferents in the ventricular and cystic striatum. The BC-cVEMP experimental approach utilized in this study is informed by the established methods of Curthoys et al. (10, 11) and Yang et al. (12). Detailed execution steps employing a bone-conduction vibrator are comprehensively illustrated in Figure 2, providing an insightful overview of our practical methodology. These evaluations aim to assess alterations in saccular and utricular function, aligning with the occurrence of hydrops in these areas in Meniere’s disease, often leading to their dysfunction (18). Vestibular organs are notably more responsive to linear acceleration, making bone conduction stimulation significantly more sensitive than air conduction stimulation. Curthoy et al. (19). similarly demonstrated the greater sensitivity of bone conduction compared to air conduction in animal experiments. Each guinea pig in this study exhibited a response to the stimulation. There’s a prevailing belief among scholars that the vestibular organs stimulated by bone conduction differ from those stimulated by air conduction. Miyamoto et al. (20) suggested that bone conduction vibration primarily stimulates the saccule and utricle, and VEMP reflects the functional status of the ipsilateral saccule and inferior vestibular nerve.

In clinical VEMP assessment, emphasis is placed on the amplitude, particularly the rectified amplitude. This parameter is crucial as VEMP amplitude is directly impacted by the extent of sternocleidomastoid muscle tension. By utilizing the rectified amplitude, the influence of muscle tension on the outcomes is mitigated, enabling a more accurate comparison of amplitude differences across diverse individuals (18–21). Studies by Murofushi et al. (21), Johnson et al. (22), and Scarpa et al. (23), revealed that patients with Meniere’s disease exhibited minimal latency prolongation, reduced amplitude, or lacked a response in cVEMPs. Some researchers suggest that varying stages of Meniere’s disease display vestibular nerve tissue degeneration (24), resulting in differences in VEMPs’ latency and amplitude. Akkuzu et al. (25) noted prolonged latency in cVEMPs associated with Meniere’s disease. Additionally, Manzari et al. (26) reported a slight amplitude increase in cVEMPs following Meniere’s attacks.

In our study, we observed a significantly higher N1 latency in the DDAVP group compared to the normal control group. Similarly, the P1 latency in the DDAVP group showed a significant increase compared to both the normal control group and the Zexie group. These findings align closely with the results reported by Johnson et al. (22) According to some researchers, the extended latency of cVEMP in Meniere’s disease might be attributed to the notable dilation of the saccule, influencing sound wave transmission. Our findings regarding amplitude indicate that the DDAVP group exhibited lower values compared to the normal control group, while the normal control group also showed higher values than the Double Zexie group. The corrected amplitude exhibited a statistically significant difference, with the normal control group showing higher values compared to the DDAVP group, Zexie group, and Double Zexie group. Some experts believe that cVEMP is an inhibitory potential that can only be measured during muscle contraction, indicating that the degree of muscle contraction significantly affects the amplitude of cVEMP (27). The underlying pathology of Meniere’s disease, EH, can influence saccular function. Research conducted by Oz et al. (28) indicated abnormal variations in cVEMP amplitude corresponding to the extent of saccular lesions. During the initial phases of Meniere’s disease, VEMP amplitude might rise in less severe saccular lesions, but significantly decrease in more advanced stages with severe saccular damage. Thus, this experiment’s results indicated that the guinea pig model induced with EH through DDAVP showcased compromised vestibular function. Upon a 7-day treatment with Zexie decoction, a notable enhancement in the vestibular function of the guinea pig model with EH was observed. The improvement displayed by the Double Zexie group was relatively more pronounced, although it did not entirely nullify the effects induced by DDAVP. As a result, the enhancement in vestibular function was somewhat diminished.

Otoacoustic emission (OAE) serves as a non-invasive, objective, and rapid detection method. It was initially discovered by British scholar D.T. Kump in 1978, revealing the ear’s capability to actively emit sound waves (29). Numerous studies have consistently highlighted that Otoacoustic Emissions (OAE) directly result from the active mechanical movement of healthy outer hair cells, thereby acting as a reliable indicator of their functional integrity. Any compromise or damage to these outer hair cells can lead to a significant reduction or even absence in OAE amplitude (30, 31). Additionally, research has demonstrated the high sensitivity of otoacoustic emissions in detecting cochlear hydrops and associated phenomena (32). ABR, an objective electrical response audiometry, serves as a vital indicator of peripheral auditory neuron function. It effectively reflects the status of the cochlea, posterior cochlear nerve, and the auditory pathway in the brainstem. Particularly beneficial for individuals unable to cooperate in behavioral test (33), ABR comprises seven waves generated across the auricular nerve, cochlear nucleus, olivary complex, lateral lemniscus, and inferior colliculus (34). In Meniere’s disease, early-stage hearing loss primarily affects low frequencies, progressing to involve both low and high frequencies in the middle stage. Subsequently, severe damage extends across all frequencies in the advanced stages (35). In this study, during the initial establishment of the model for seven consecutive days, the latency of waves I-V in the normal control group demonstrated significant differences compared to the DDAVP and Zexie groups. Additionally, the Double Zexie group exhibited lower latency of waves I-V than both the Zexie and DDAVP groups, with statistically significant variances. Following the establishment of the model for 14 consecutive days, waves I-IV latency in the normal control group showed marked differences compared to the Zexie group. Moreover, waves I and II latency in the normal control group was significantly lower than that in the DDAVP group. Notably, waves I-V latency in the Double Zexie group was notably lower than in both the Zexie and DDAVP groups, with waves III, IV, and V latency in the normal control group being significantly lower than in the Double Zexie group, all indicating statistical significance. These findings suggest that Zexie decoction exhibits a degree of efficacy in alleviating the induced hearing impairment by DDAVP in guinea pigs with EH. The extent of this amelioration correlates with the concentration and dosage of Zexie decoction. However, even at a doubled dosage, Zexie decoction only partially mitigated the vestibular and auditory functional decline caused by DDAVP, falling short of complete counteraction. Furthermore, the efficacy of Zexie decoction diminished with the prolonged application time of DDAVP. We analyzed the reason for this, which may be related to the degeneration of vestibular nerve tissue in patients with Meniere’s disease (24), therefore, the latency of the DDAVP group was longer than that of the control group. In conclusion, the above experimental results show that Zexie decoction can partially improve the decline of vestibular and auditory functions caused by DDAVP, but it cannot completely offset it. Moreover, with the prolongation of DDAVP application, the improvement of Zexie decoction also weakens.

Scanning electron microscopy (SEM) analysis revealed that at 7 days, the outer hair cells exhibited less damage in both the Zexie decoction group and the double-dose Zexie decoction group, although ciliary displacement and loss were still present, with slightly milder damage observed in the double-dose group. Following 14 days of continuous treatment, both the Zexie decoction and double-dose groups showed reduced outer hair cell damage compared to the desmopressin (DDAVP) group, yet significant ciliary displacement and loss were evident. The double-dose group displayed less damage compared to the single-dose group but still experienced considerable outer hair cell loss compared to the 7-day assessment. This suggests that the severity of outer hair cell damage associated with DDAVP-induced endolymphatic hydrops is substantial and challenging to completely reverse. Historically, there have been no reports from scanning electron microscopy studies on the state of hair cells in guinea pig models of membranous labyrinth hydrops. This study, from a morphological perspective, investigates the condition of outer hair cells during treatment with Zexie decoction for DDAVP-induced endolymphatic hydrops in guinea pigs. After 7 days of treatment, Zexie decoction was found to offer protective effects on hair cells, with the double dosage providing stronger protection. However, as the modeling duration increased to 14 days, the protective efficacy of the double dosage of Zexie decoction diminished. This also demonstrates that Zexie decoction can improve vestibular and auditory functions in the DDAVP-induced EH guinea pig model, although the improvement decreases with prolonged DDAVP administration.

Despite the valuable insights gained from this experiment, certain limitations warrant further investigation, especially in clinical settings. Ethical approval notwithstanding, factors like patient age, overall health condition, disease duration, hearing loss severity, and frequency of vertigo attacks could impact experimental data. Hence, a broader clinical dataset is essential for robust validation. While numerous experiments have linked Meniere’s disease to *in vivo* hormone metabolism disorders, hypotheses involving inner ear immunity and viral infections persist. Therefore, this experiment demonstrates outcomes specific to the EH model induced by DDAVP. Endolymphatic hydrops is a characteristic pathological manifestation of Meniere’s disease, and the mechanisms of hydrops that have been discovered so far may include overproduction or decreased endolymphatic absorption, and increased activity of aquaporins. Among aquaporins, AQP2 is crucial, and in the inner ear, AQP2 is widely distributed in places such as basement membrane, endolymphatic sac, spiral ganglia, etc. (36, 37) It has also been shown that the expression of AQP2 mRNA in the cochlea increases with the increase of vasopressin(AVP) receptor expression, and the activation of the AVP receptor can significantly improve the reabsorption of free water, which proves that AVPR-AQP2 plays an important role in the fluid regulation of the inner ear. In our previous study, we found that DDAVP can activate not only the classical cAMP-PKA signaling pathway but also the cAMP-Epac pathway, thereby regulating the expression of AQP2 (6, 7). Some studies have pointed out that Zexie decoction can regulate AQP2 through the cAMP-PKA pathway, reducing water permeability and regulating the fluid balance in the inner ear. However, it is still unknown whether Zexie decoction can also regulate AQP2 and exert a diuretic effect through the cAMP-Epac pathway. Moving forward, our research would aim to delve into the precise active constituents of Zexie decoction and their molecular-level impact on improving the structure and function of vestibular and cochlear hair cells. Additionally, we intend to explore its comprehensive effects on vestibular and auditory nerve pathways. The subsequent foundational steps include identifying specific biologically active components through drug purification techniques. Initially, we plan to administer the drug systemically and then measure its concentration in both serum and perilymph, to understand the pharmacokinetic characteristics of the drug in the blood and the inner ear. This will aid in comprehending the drug’s capability to cross the blood-labyrinth barrier. Following this, we intend to administer the drug via tympanic injection and measure its concentration in the perilymph to assess whether this method can achieve higher drug concentrations in the inner ear. This step is crucial for further validating whether an optimized route of administration can lead to more effective treatment outcomes. We anticipate achieving a more potent protective effect upon uncovering these specifics.

## Conclusion

The outcomes obtained from BC-cVEMP, ABR, scanning electron microscopy and DPOAE analyses indicated a positive impact of Zexie decoction on the vestibular and auditory functions in guinea pig models with EH induced by DDAVP. Notably, this improvement demonstrated a correlation with the concentration and dosage of Zexie decoction. However, even with a double dose, Zexie decoction only exhibited partial efficacy in ameliorating the vestibular and auditory impairments induced by DDAVP, unable to completely counteract its effects. Moreover, as the duration of DDAVP application extended, the ameliorative effects of Zexie decoction diminished.

## Data Availability

The original contributions presented in the study are included in the article/Supplementary material, further inquiries can be directed to the corresponding author/s.
